# Disrupted metabolic and spontaneous neuronal activity of hippocampus in sepsis associated encephalopathy rats: A study combining magnetic resonance spectroscopy and resting-state functional magnetic resonance imaging

**DOI:** 10.3389/fnins.2022.1032098

**Published:** 2022-11-17

**Authors:** Haojia Li, Hongsen Liao, Chen Zhang, Yajie Xu, Xiaomin Xu, Yuchen Chen, Shaozheng Song, Qian Li, Yanna Si, Hongguang Bao

**Affiliations:** ^1^Department of Anesthesiology, Nanjing First Hospital, Nanjing Medical University, Nanjing, Jiangsu, China; ^2^Department of Radiology, Nanjing First Hospital Nanjing Medical University, Nanjing, Jiangsu, China; ^3^Department of Basic Medicine, School of Health and Nursing, Wuxi Taihu University, Wuxi, Jiangsu, China; ^4^Department of Anesthesiology, Jiangning Hospital Affiliated to Nanjing Medical University, Nanjing, Jiangsu, China

**Keywords:** cecal ligation and puncture (CLP), hydrogen proton magnetic resonance spectroscopy (1H-MRS), neural inflammation, resting-state functional magnetic resonance imaging (rs-fMRI), sepsis-associated encephalopathy (SAE)

## Abstract

**Background:**

The diagnosis of sepsis associated encephalopathy (SAE) remains challenging in clinical settings because of a lack of specific biomarkers. Functional magnetic resonance imaging (fMRI) and proton magnetic resonance spectroscopy (1H-MRS) can be used to aid in the diagnosis of cognition related diseases. This study investigated changes in functional activities and brain metabolites in the hippocampus in SAE rats by fMRI and 1H-MRS.

**Materials and methods:**

Sepsis associated encephalopathy rats underwent cecal ligation and perforation (CLP) surgery. The Morris water maze (MWM) test was then used to evaluate cognitive function. Resting state-fMRI and 1H-MRS scanning were performed 7 and 14 days after CLP surgery to reveal spontaneous neuronal activity and metabolite changes in the hippocampus. The amplitude of low-frequency fluctuation (ALFF) was used to evaluate spontaneous neuronal activity in the hippocampus. Creatine (Cr), Myo-inositol (mI), and glutamine/glutamate (Glx) levels were measured with 1H-MRS scanning. Immunofluorescence and levels of interleukin (IL)-1β, interleukin (IL)-6, and C-reactive protein (CRP) in the hippocampus were additionally detected to evaluate microglial mediated inflammatory responses. Statistical analysis was performed to evaluate correlations between hippocampal metabolism and behavioral findings.

**Results:**

Cecal ligation and perforation treated rats exhibited impaired learning and memory function in the MWM test at days 7 and 14. Elevation of IL-1β in the hippocampus, as well as immunofluorescence results, confirmed severe neuro inflammation in the hippocampus in SAE rats. Compared with the sham group, the ALFF of the right CA-1 area of the hippocampus was higher at day 7after CLP surgery. The Glx/Cr and mI/Cr ratios were enhanced at day 7 after CLP surgery and slightly lower at day 14 after CLP surgery. The ALFF value, and Glx/Cr and mI/Cr ratios were negatively correlated with time spent in the target quadrant in the MWM test.

**Conclusion:**

Spontaneous neuronal activity and metabolites showed significant alterations in SAE rats. The elevated ALFF value, Glx/Cr ratio, and mI/Cr ratio in the hippocampus were positively associated with cognitive deficits. Changes in ALFF and metabolites in hippocampus may serve as potential neuroimaging biomarkers of cognitive disorders in patients with SAE.

## Introduction

Sepsis-associated encephalopathy (SAE) is one of the most serious complications of sepsis characterized by delirium and cognitive dysfunction (Tauber et al., 2021). More than 50% of surviving patients with sepsis suffer from severe and long-term cognitive deficits (Chung et al., 2020). In general, neuroinflammation, metabolic disorders, and depressed spontaneous neuronal activity have been considered to be causative factors for cognitive impairment in SAE patients (Peng et al., 2021). Studies have shown that the levels of inflammatory cytokines, such as interleukin (IL)-1β, interleukin (IL)-6, and tumor necrosis factor α, increase significantly in the hippocampus of SAE rats, accompanied by microglial activation (Tauber et al., 2021). But the research in metabolites and excitability of neurons in SAE patients by non-invasive and objective methods was limited.

Recently, neuroimaging studies investigated brain functional and metabolic changes with functional magnetic resonance imaging (fMRI) and hydrogen proton magnetic resonance spectroscopy (1H-MRS) in various neurodegenerative diseases (Piersson et al., 2021). Resting-state fMRI (rs-fMRI) detected the blood oxygenation level dependent (BOLD) signal, and provided information on regional spontaneous neuronal activity with the amplitude of low-frequency fluctuation (ALFF), which is the most commonly used analysis method for fMRI (Zou et al., 2008; Huang et al., 2021). A decreased ALFF values in the right hippocampus was shown to be directly related to the deterioration cognitive function in mild cognitive impairment patients (Tao et al., 2019). However, more researches were needed to investigate the trends of ALFF value in hippocampus of SAE rats. Besides rs-fMRI imaging analysis, 1H-MRS has been shown to be an accurate tool for gaining insights into brain metabolic function. Metabolic alterations which were detected by 1H-MRS provide valuable information on cerebral diseases (Hsu et al., 2021). Myo-inositol (mI) indicated microglial-induced neuroinflammation in the brain (El-Abtah et al., 2022 Feb), while the increased concentration of glutamate/glutamine (Glx) was found in patients with hepatic encephalopathy (Kuan et al., 2021). To date, whether these metabolic alterations present in the hippocampus of SAE rats remains unclear. Further research is needed on whether these metabolic alterations are due to sepsis and can be correlated with cognitive related behaviors.

Since sepsis is a heterogeneous and complex pathology, *in vivo* models are valuable tools to explore its underlying mechanisms. The cecal ligation and puncture (CLP) surgery yields reliable outcomes of sepsis associated encephalopathy in rats (Gao et al., 2021). Previous studies have found that serious inflammation of the hippocampus lasted 15 days post CLP surgery, and that the result of cognitive related behavior tests performed 1–2 weeks post CLP surgery indicated a significant cognitive decline in SAE rats (Li et al., 2020). The hippocampus participates in mediating cognition and emotion. Our previous studies suggest that abnormal hippocampal inflammation is an essential pathogenic trait of SAE (Cai et al., 2021). Neuro inflammation in the hippocampus caused by SAE may lay the groundwork for the cognitive dysfunction. However, it remains unknown the relationship between neuro inflammation and metabolic alterations, as well as impairs cognitive function in SAE rats.

In the present study, we examined brain ALFF and metabolites changes in the hippocampus of SAE rates during 7–14 days post CLP surgery by rs-fMRI and1H-MRS. We further investigated the relationship between Morris water maze tests and rs-fMRI results at 7 and 14 days post CLP surgery in SAE rats. The serum levels of IL-1β and IL-6, as well as immunofluorescence, were also analyzed at the same time points. The objective of this research is to investigate the features of ALFF and metabolite changes in hippocampus of SAE rats, and to further explore its relationship with impairment of cognitive functions in SAE rats. Our findings may provide potential reference for comprehension of the neural mechanisms underlying SAE.

## Materials and methods

### Animal model

All animal procedures were approved by the Institutional Animal Care and Use Committee of Nanjing Medical University (IACUC: 2103051; Animal Use Permit SYXK(Su)2020-0022). In total 24 adult male Sprague Dawley rats (Nanjing, Jiangsu Laboratory Animal Center, China; 8 weeks, 280–320 g) were divided into three groups, sham group, day 7 post CLP group and day 14 post CLP group. The room in which the rats were housed was maintained at 22°C with a 12-h light–dark cycle. The animals were treated humanely with free access to food and water with regard for alleviation of suffering.

For surgical procedures, rats were anesthetized by pentobarbital sodium at a dose of 35 mg/kg intraperitoneally (i.p.) according to previous research (Rittirsch et al., 2009). CLP surgery was performed on 16 rats to induce sepsis. Briefly, under aseptic conditions, a 3 cm midline laparotomy was performed to allow exposure of the cecum with adjoining intestine. The cecum was then ligated tightly with a 3.0 silk suture at its base under the ileocecal valve and punctured twice with a 22-gauge needle. The cecum was then gently squeezed to extrude a small amount of feces from the perforation site. The cecum was returned to the peritoneal cavity, and the laparotomy incision was closed with 4-0 polyglactin sutures. Following surgery, fluid losses were replenished by administration of 5 ml/100 g of warm (37°C) isotonic saline i.p., and the rats were placed in their cages. In the sham group, under aseptic conditions, only laparotomy was performed, and the cecum was neither ligated nor punctured. All animals displayed signs of encephalopathy at 24 h post CLP surgery (lethargy, mild ataxia, lack of spontaneous movement).

### MRI acquisition

Following CLP surgery, all animals were subjected to MRI of their brain, 7 days and 14 days post-CLP surgery. MRI scans were performed with a 7 Tesla horizontal bore magnet (Bruker Biospec 7T/20 USR; Bruker, Karlsruhe, Germany). Accurately 2% isoflurane in oxygen and air was used to produce anesthesia. The rats were then placed in a prone posture on a small animal MRI scan bed and their heads were fixed with dental hooks and ear bars to reduce head movements. Throughout the scanning period, the degree of anesthesia was maintained by modulating the isoflurane concentration (2–2.5%) to preserve the respiration rate of 80–100 breaths per minute and oxygen saturation of 95–100%. Magnetic resonance scanning was started after the respiration, heart rate and saturation of rats were observed to be stable. Fast spin-echo T2 weighted MR images were obtained for visualizing the structure of the brains with the following parameters: slice = 25, repetition time (TR)/echo time (TE) = 3000/33 ms, FA = 90°, TA = 4 min 48 s, SI = 1.00/1.00 mm, FOV = 3.17/2.50 cm, image size = 256. Functional images were obtained using BOLD sequence, TR/TE = 2000 ms/25 ms, slices = 18, slice thickness = 1 mm, TA = 5 min, image size = 128 × 128, flip angle = 90°, repetition = 150, FOV = 28.80/18.00 mm, Image resolution = 128 × 128.

### Resting-state functional magnetic resonance imaging data preprocessing and analysis

The first ten volumes of rs-fMRI data were discarded for MRI signaling to reach a steady state. Then, the images were realigned in a time series. Those on which the head moved by more than 0.2 mm and 2 degrees were excluded. We first magnify the BOLD image by 10 times (Suzuki et al., 2013), and then the BOLD images were registered to T2 structure images of each rat by non-linear transformation. Following, the images were registered to Wistar rat brain templates (resampling voxel size = 3 mm × 3 mm × 3 mm) using the Statistical Parametric Mapping package in SPM8.^1^ Next, the fMRI image voxels were spatially normalized to 0.3 × 0.3 × 0.3 mm and smoothed with an isotropic Gaussian kernel (FWHM = 0.4 mm) as actual sizes of rats. The time series for each voxel was transformed to the frequency domain and the power spectrum was then obtained. The square root was calculated at each frequency of the power spectrum and the average square root was obtained across 0.01–0.08 Hz at each voxel. This averaged square root was taken as the ALFFs were normalized by the mean within-brain ALFF value for each subject with DPABI software.^2^ We performed an ANOVA among the three groups (sham vs. day 7 post CLP vs. day 14 post CLP) using the toolbox in SPM8. After significant clusters were generated within a binary mask, *post-hoc* two-sample *t*-tests were performed between the two experimental groups within the previously generated mask. All significance tests were conducted at an FDR threshold of *P* = 0.005. We abstracted right CA-1 area from ANOVA analysis results, and the ALFF values of the right CA-1 area of hippocampus were extracted from mean within-brain ALFF maps. These ALFF values were used as biomarker to evaluate brain function of rats.

### Hydrogen proton magnetic resonance spectroscopy acquisition and analysis

Hydrogen proton magnetic resonance spectroscopy scans were performed after fMRI scan using 7 Tesla MRI scans. A Fast Spin Echo T2W image was performed to position 1-H MRS voxels of the hippocampus using an ultrashort echo time (TR/TE = 2500/16.6 ms) spectroscopy method. The target voxel of interest = 2 × 2 × 1 mm^3^ with a TA = 5 min 20 s. After extracting MR spectra in these groups, we analyzed and quantified the curve using Mestrenova software.^3^ The compound was located by the position of the curve crest, N-acetylaspartate (NAA) located at 2.02ppm, creatine (Cr) located at 3.05 ppm, choline-compound (Cho) located at 3.20 ppm, Myo-inositol (mI) located at 3.56 ppm, lipid (Lip) located at 1.30 ppm and glutamine/glutamate (Glx) located at 3.75 ppm. The areas under a peak of NAA, Cr, Cho, mI, and Glx were measured, and the Cho/Cr, Glx/Cr, mI/Cr, NAA/Cho, and Lip/Cr ratios were determined.

### Hippocampus-dependent behavioral tasks

Cognitive functions of spatial learning and memory were evaluated in the three groups rats by MWM test according to the methods of Wang et al. (2017). The diameter of the pool and the platform were 180 and 10 cm, respectively, and the platform was placed 2 cm below the water. The temperature of water was kept range from 22 to 24°C. The tests were recorded by a camera that was hung above the pool and the results were analyzed by ANY maze Video Tracking System (Stoelting Co., Wood Dale, IL, USA). The rats in the sham group and day 7 post CLP group were subjected to the MWM during day 3–7 post CLP surgery, while the rats in the day 14 CLP group were subjected to the MWM during day 10–14 post CLP surgery. During the continuous 4 days of training, each rat was released into the pool to find a platform placed under the water. This was performed four times from every different quadrant each day. The escape latency was recorded for each training session. Space exploration training was performed at the end day of the MWM test. The platform was removed, and all rats were monitored for 60 s in order to observe the time spent in the target quadrant and frequency of crossing the platform.

### Enzyme-linked immunosorbent assay

The hippocampus was removed after the rats were sacrificed, lysed with radioimmunoprecipitation assay (RIPA) buffer (Abcam, ab156034, Cambridge, UK), homogenized and centrifuged at 20,000 g for 1 min at 4°C. The levels of IL-1β, IL-6, and CRP were determined by ELISA kits (Solarbio, Beijing, China) according to the manufacturer’s instructions, and the absorbance was measured with a micro-plate reader at 450 nm [optical density(OD) value].

### Immunofluorescence

The rats were injected i.p. with pentobarbital sodium, and the brains harvested at 7 and 14 days post-surgery. Brains were fixed in 4% paraformaldehyde for 24 h at room temperature, embedded in paraffin, and cut into 5-μm coronal sections. The sections were blocked with 1% bovine serum albumin and incubated overnight at 4°C with a primary antibody against Iba-1 (1:100; Abcam, ab178846, Cambridge, UK), and then with a goat anti-rabbit IgG H&L (Cy3) (1:100, Abcam, ab6939) for 1 h at 4°C. Sections were then counterstained with 4,6-diamidino-2-phenylindole for 10 min at room temperature. Images were taken using a servicebio fluorescence microscope (Pannoram) and the fluorescence intensity of Iba-1 was analyzed using ImageJ software (National Institutes of Health, Bethesda, MD, USA).

### Statistical analysis

Data are presented as mean ± SD was analyzed and were analyzed using SPSS 22.0 (SPSS Inc., Chicago, IL, USA) and GraphPad Prism 8 (GraphPad Software, San Diego, USA). Behavior test results, ALFF values, metabolism concentrations and ELISA results were analyzed using one-way ANOVA, followed by Tukey’s honest significant difference test. Statistical significance was accepted at *p* < 0.05. The relationships between ALFF values, metabolism concentrations, and total time spent in the target quadrant in MWM were explored using Spearman’s correlation analysis.

## Results

### Cecal ligation and perforation surgery induced amplitude of low-frequency fluctuation value abnormalities in sepsis associated encephalopathy rats

**FIGURE 1:**
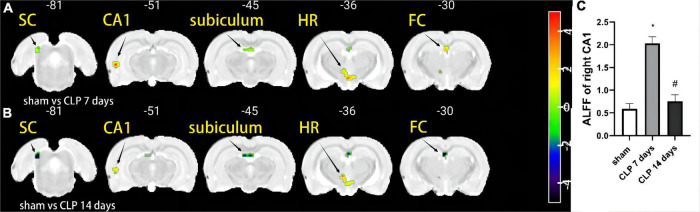
The amplitude of low-frequency fluctuation (ALFF) differences in the day 7 post cecal ligation and perforation (CLP), and day 14 post CLP groups. Warm (red-yellow) colors represent increased and cold (blue-green) colors decreased ALFF values in the day 7 post CLP, and day 14 post CLP groups. **(A)** ALFF values were higher in the right CA-1, left FC, and HR in the day 7 post CLP group. **(B)** The left FC, right deep layers of the SC, CA-1, and subiculum showed lower ALFF in the day 14 post CLP group (*n* = 8 in each group. FDR corrected *p* < 0.005, cluster size > 10 voxels). **(C)** The ALFF value of right CA-1 was significantly higher in the day 7 post CLP group than sham group, but lower in day 14 post group (*n* = 8, *p < 0.05, compared with sham group, ^#^*p* < 0.05, compared with day 7 post CLP group, analyzed by one-way ANOVA). CA-1: Cornu ammonis-1; SC: deep layers of the superior colliculus; FC: fasciola cinereum; HR: hypothalamic region.

After CLP surgery intervention, compared with the sham group, the ALFF values in the day 7 post CLP group were higher in the right cornu ammonis-1 (CA-1) area, left fasciola cinereum (FC), and hypothalamic region (HR) compared with sham group (*p* < 0.005, FDR corrected, *n* = 8, Figure 1A). The ALFF values were lower in the left FC, right deep layers of the superior colliculus (SC) and subiculum in the day 14 post CLP group (*p* < 0.005, FDR corrected, *n* = 8, Figure 1B). The ALFF value of right CA-1 followed the rule of “enhanced-decreased” at day 7 and 14 post CLP surgery (Figure 1C). The number of voxels and Montreal Neurological Institute (MNI) coordinates of the abnormal brain regions in rats of CLP groups were listed in Table 1.

**TABLE 1:** Regions showing amplitude of low-frequency fluctuation (ALFF) differences among the sham, day 7 post cecal ligation and perforation (CLP), and day 14 post CLP groups.

Location cluster (AAL)	Number of voxels	MNI coordinates			Peak F
		*x*	*y*	*z*	
Hypothalamic region L	14	2.0	35.1	14.8	40.1
Cornu ammonis 1 R	10	65.0	50.1	9.2	34.2
Deeper layers of the superior colliculus R	13	29.0	80.1	36.2	80.5
Subiculum R	10	–1.0	44.1	36.2	55.5
Fasciola cinereum L	12	2.0	29.1	39.2	55.4

### Altered brain metabolism in the hippocampus post cecal ligation and perforation surgery

**FIGURE 2:**
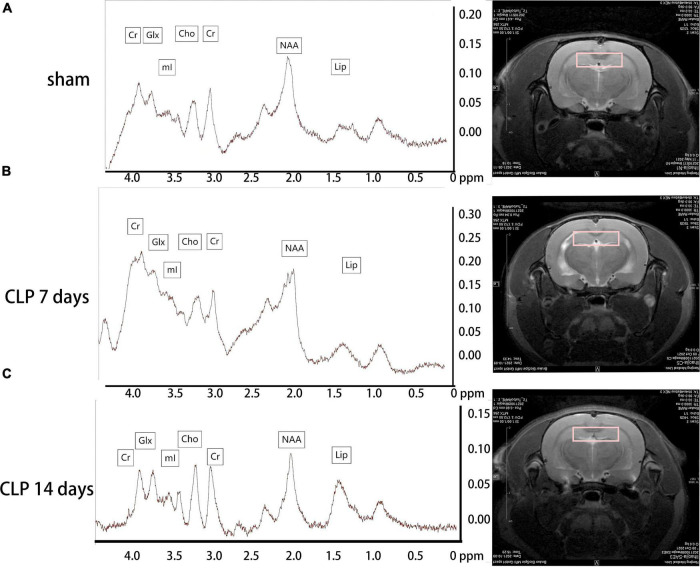
Metabolites in the hippocampus detected by 1H-MRS. Metabolites in the hippocampus were analyzed by 1H-MRS, and the spectrums determined by MestReNova. The representative metabolite spectrum in the hippocampus of rats and region position in the coronal image in the sham group **(A)**; in the day 7 post CLP group **(B)**; in the day 14 post CLP group **(C)**.

**FIGURE 3:**
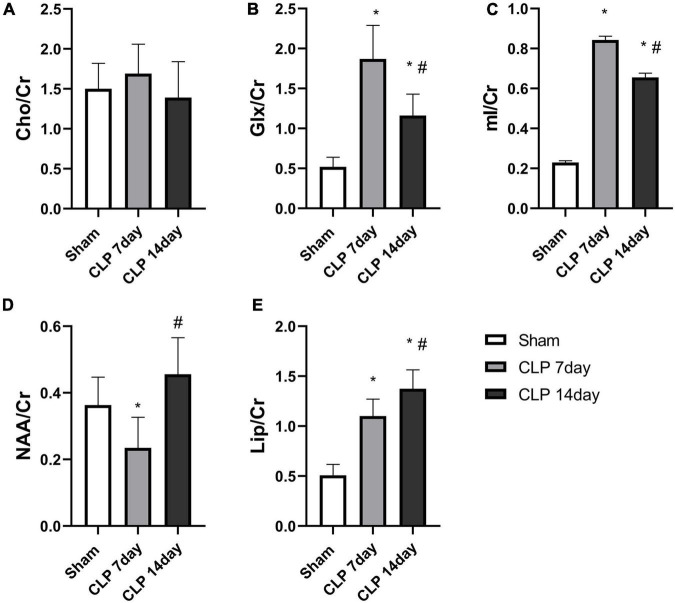
Metabolite analysis among the three groups. There was no obvious difference in the **(A)** Cho/Cr ratio among the groups (*p* > 0.05, analyzed by one-way ANOVA, *n* = 8); The Glx/Cr **(B)** and mI/Cr ratios **(C)** were significantly increased in the hippocampi of day 7 post CLP group, but were obviously lower in day 14 post group. **(D)** NAA/Cr ratios in the right hippocampus generally followed the rule of “decreased-enhancement” post CLP surgery. **(E)** Lip/Cr ratios increased gradually post CLP surgery (*p < 0.05, compared with sham group, ^#^*p* < 0.05, compared with day 7 post CLP group, analyzed by one-way ANOVA) (*n* = 8. Data are expressed as the mean ± SD).

Metabolites in the hippocampus were detected by 1H-MRS, and the spectrums are shown in Figure 2. There was no significant difference among the three groups in the Cho/Cr ratio (*p* > 0.05, Figure 3A). Significantly higher Glx/Cr and mI/Cr ratios were observed on day 7 and 14 post CLP surgery. Compared with the day 7 post CLP group, the Glx/Cr and mI/Cr ratios were significantly lower on day 14 post CLP group (*p* < 0.05, Figures 3B,C). The NAA/Cho metabolite ratio was significantly lower in the day 7 post CLP group than in sham group (*p* < 0.05). However, NAA/Cho ratio in the day 14 post CLP group was higher than that in day 7 post CLP group (*p* < 0.05, Figure 3D). The ratio of Lip/Cr rose continuously after CLP surgery, compared with sham group (*p* < 0.05, Figure 3E).

### Cecal ligation and perforation surgery enhanced microglia related inflammation in sepsis associated encephalopathy rats

**FIGURE 4:**
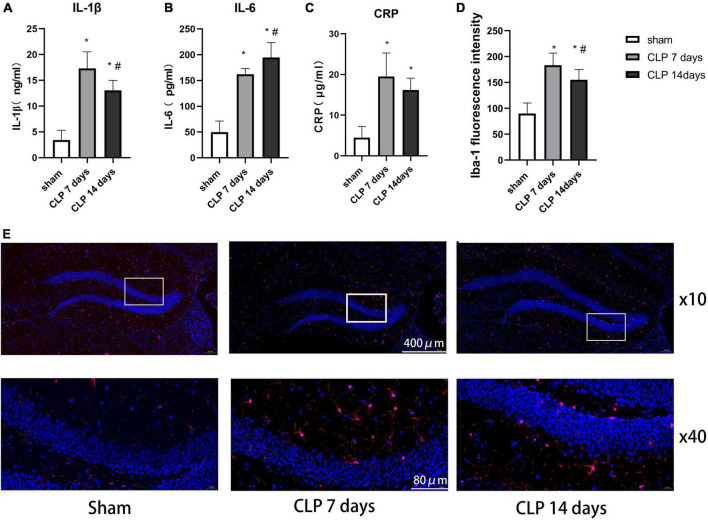
Cecal ligation and perforation surgery enhanced microglia related inflammation in hippocampus of SAE rats. The difference in panel **(A)** IL-1β, **(B)** IL-6, and **(C)** CRP levels in hippocampus among the three groups. **(D)** The intensity of Iba-1 in the hippocampus detected by immunofluorescence staining (*p < 0.05, compared with the sham group, ^#^*p* < 0.05, compared with day 7 CLP group, analyzed by one-way ANOVA, *n* = 8). Data are expressed as the mean ± SD. **(E)** Representative immunofluorescence staining of Iba-1 (red) in the hippocampus, *n* = 8 per group.

We examined the expression of IL-1β (Figure 4A), IL-6 (Figure 4B), and CRP (Figure 4C) to assess the severity of inflammation in hippocampus of SAE rats. It was observed that IL-1β, IL-6, and CRP levels in hippocampus of SAE rats were notably higher than those in the sham group (*p* < 0.05). The expression of IL-1β in the day 14 post CLP group were significantly lower than those in the day 14 post CLP group (*p* < 0.05). However, the expression of IL-6 was higher in the day 14 post CLP group than that in day 7 post CLP group (*p* < 0.05). No significant difference in CRP levels was found between the day 7 and 14 post CLP groups.

Ionized calcium binding adaptor molecule-1 (Iba-1) immunofluorescence staining was performed to detect the microglial distribution in the hippocampus (Figure 4E). In the day 7 post CLP groups, the fluorescence intensity of Iba-1 increased significantly than that in the sham group, but decreased in day 14 post CLP group compared with day 7 post CLP group (Figure 4D). These results indicated that Iba-1-positive cells gathered in the hippocampus after CLP surgery.

### Cecal ligation and perforation surgery deteriorates cognitive performance in the Morris water maze task

**FIGURE 5:**
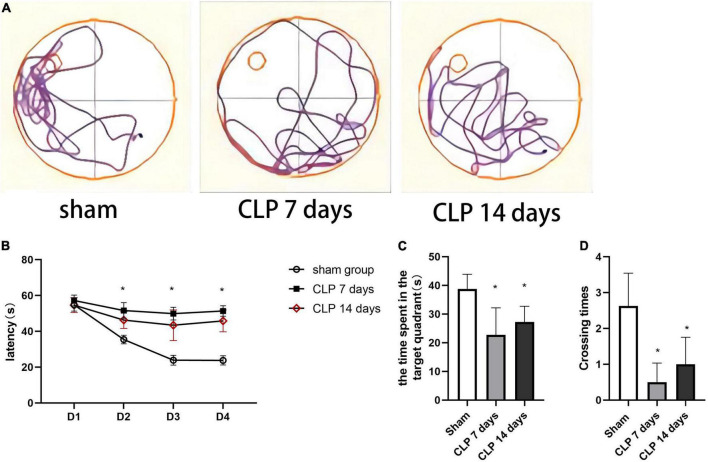
Cecal ligation and perforation surgery deteriorates cognitive performance in the MWM task. **(A)** The typical tracks of rats in the three groups in the probe test. **(B)** The latency of finding the hidden platform in on training days (*p < 0.05, versus sham group, *n* = 8) analyzed by repeated measures ANOVA and Tukey’s *post hoc* comparison. **(C)** The time spent in the target quadrant in the probe trail and **(D)** the number of crossings the platform location (*p < 0.05, versus sham group, *n* = 8) analyzed by one-way ANOVA. Data are expressed as the mean ± SD.

To evaluate CLP-induced memory impairment over time, we studied the learning and memory performance of rats at 7 and 14 days after CLP surgery by MWM test. The tracks of rats in probe tests of MWM were shown in Figure 5A. During the training session of MWM, the rats in day 7 post CLP group exhibited decreased in learning function, as was demonstrated by increased latency to platform. However, the platform latency changed little in day 14 post CLP group compared with that in day 7 post CLP group (Figure 5B). In the space exploration experiment, memory performance of the rats was analyzed in terms of the time spent in the target quadrant of MWM test. CLP treated rats exhibited decreased duration in the target quadrant at 7 and 14 days (Figure 5C). Furthermore, CLP surgery decreased the number of crossing platform at 7 and 14 days post CLP (Figure 5D). However, the differences in the time spent in the target quadrant and the times of crossing the platform were not significantly between 7 and 14 post CLP groups. The results suggested that the learning and memory functions of CLP treated groups were impaired from 7 to 14 days after CLP surgery in the Morris water maze test.

### The correlation analysis between the amplitude of low-frequency fluctuation values, metabolites, and behavioral parameters

**FIGURE 6:**
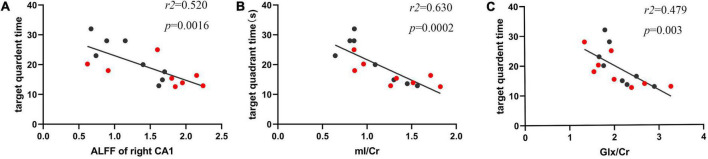
The ALFF values and metabolites in the hippocampus were negatively correlated with duration in the target quadrant. **(A)** The ALFF value of the right CA-1 was negatively correlated with total time spent in the target quadrant (*r*^2^ = 0.520, *p* = 0.0016). **(B)** The mI/Cr ratio was negatively correlated with target time spent in the target quadrant (*r*^2^ = 0.630, *p* = 0.0002). **(C)** The Glx/Cr ratio was negatively correlated with total time spent in the target quadrant (*r*^2^ = 0.479, *p* = 0.003). The red samples represented the rats from day 7 post CLP group, and black samples from day 14 post CLP group.

As we found that the ALFF values and Glx/Cr and mI/Cr ratios fluctuated post CLP surgery, we evaluated the correlation between ALFF values of the CA-1 region with Glx/Cr and mI/Cr ratios and behavior test results. The ALFF value of the right CA-1 was negatively correlated with the total time in spent in the target quadrant, which reflected the capacity of episodic memory (Figure 6A; *r*^2^ = 0.520, *p* = 0.0016). We also analyzed the correlation between metabolites and time spent in the target quadrant. The mI/Cr ratio was negatively correlated (Figure 6B; *r*^2^ = 0.630, *p* = 0.0002), whereas the Glx/Cr ratio was negatively correlated (Figure 6C; *r*^2^ = 0.479, *p* = 0.003), with time spent in the target quadrant.

## Discussion

In this study, we measured the ALFF value and metabolite changes in the hippocampus during the progression of sepsis associated cognitive impairments 7 and 14 days post CLP surgery. We found that the ALFF values in the right CA-1 area of the hippocampus was higher at day 7 post CLP surgery than sham group, and lower at 14 days post CLP surgery. Conversely, the Glx/Cr and mI/Cr ratios were higher at day 7 post CLP surgery and were lower at 14 days post CLP surgery; trends of which were coincident with IL-1β levels in the hippocampus. Furthermore, ALFF value of right CA-1, the Glx/Cr and mI/Cr ratios were negatively related to cognitive function, suggesting brain ALFF values and metabolite alterations can be used to evaluate cognitive deficits in SAE rats.

It is known that SAE is the most common cause of encephalopathy in medical-surgical ICUs. The neural metabolic disturbance sand functional deficits in the CNS are responsible for cognitive impairment of SAE patients (Xie et al., 2022). Previous studies have shown that the diagnosis of SAE relies on questionnaires such as the Confusion Assessment Method for the ICU, as well as delirium screening tools (Feng et al., 2019; Klawitter et al., 2020). Currently, rs-fMRI is gaining growing popularity in the clinical evaluation of cognitive impairment such as Alzheimer’s disease, post-traumatic stress disorder, and post-operative cognitive dysfunction (Herringa, 2017; Zhang et al., 2019), but studies on its use in SAE patients are still limited. In our study, we found significantly higher ALFF values in the right CA-1, left FC, and HR in SAE rats at 7 days post CLP surgery by rs-fMRI. Conversely, the ALFF values were lower in the left FC, right deep layers of the SC, CA-1, and subiculum at 14 days post CLP. The regions studied, including the FC, CA-1, and subiculum are important components of the hippocampus, which play a significant role in spatial learning and memory in rats. The relationship between ALFF values and cognitive function test remains to be further elucidated.

The hippocampus is well known for its role in long-term memory and MWM-associated spatial learning and memory in rodents has been linked to the hippocampus (Liu et al., 2021). As CLP surgery has been shown to cause sustained cognitive impairment 5–15 days post-surgery and a previous study performed the MWM test at 1–2 weeks post CLP surgery to assess cognitive function in SAE rats (Shen et al., 2020; Giridharan et al., 2022), we further analyzed the ALFF values and metabolism changes during the same time period. In our results, the increased ALFF values in the hippocampus supported the deteriorated behavioral results in the MWM test, which was consistent with a previous studies using the MWM test to evaluate hippocampal function (Barnhart et al., 2015). As shown in previous research, the brain functional connection between right hippocampus and thalamus increased in CLP induced SAE rats, and the enhanced functional values between the two regions were negatively correlated with behavioral performance in rats with SAE (Yao et al., 2022). These results suggested that CLP surgery not only increased regional spontaneous neuronal activity in hippocampus, but also enhanced the functional connection between hippocampus and other brain regions. This is in agreement with the findings of a previous study indicated that changes in hippocampal function biomarkers detected by fMRI might reflect the spatial memory ability of rats (Shah et al., 2019). Therefore, the ALFF values of the right CA-1 area are an excellent biomarker to evaluate the cognitive function in SAE rats.

In recent years, 1H-MRS has been widely used to detect neurochemical alterations in specific brain regions of patients with brain tumors, indicating that metabolic changes can be used as biomarkers to identify brain tumors (Liu et al., 2021). In the present study, we obtained 1H-MRS data from the hippocampi of rats in the sham and CLP surgery groups. The mI/Cr ratio, a marker for the glial cell proliferation, was significantly increased in the day 7 and 14 post CLP group, consistent with the trend of IL-1β levels in serum. Recent research demonstrated that mI can cause glial cells differentiation, and that the elevation of the mI/Cr ratio is often interpreted to reflect glial cell activation (Schnider et al., 2020). With a focus on changes in immunofluorescence staining results after CLP surgery, we observed that microglial cells activated in the hippocamp of SAE rats, which supported the hypothesis that the mI/Cr can reflect central inflammation in SAE rats. We also found the Glx/Cr ratio was elevated in the day 7 and 14 post CLP groups. Recent studies have shown that higher Glx/Cr ratio was obvious in minimal hepatic encephalopathy patients (Chen et al., 2020). The increase in the levels of intracellular glutamine leads to osmotic dysregulation and astrocyte swelling in hippocampus (Karczewska-Kupczewska et al., 2018). In addition to the findings presented above, we detected the higher mI/Cr and Glx/Cr ratios were negatively correlated with time spent in the target quadrant of MWM test. Our results demonstrate that the mI/Cr and Glx/Cr ratios are significant bio-markers in the CLP induced SAE rat model. As there is mounting evidence that differences in metabolites are evident in early SAE rats, future studies to assess cognitive decline of SAE patients by 1H-MRS are needed.

There were some limitations in our study. First, we did not distinguish between lipid and lactate, since these two metabolites show overlapping resonance frequencies (chemical shifts) at 1.33–1.35 PPM. Observations of higher Lip/Cr may reflect neuronal damage in SAE rats, a finding that awaits further investigation with newer acquisition techniques. Furthermore, we only tested the rats in at day 7 and 14 post CLP surgy by rs-fMRI and pathological methods; long-term observation should be administered in future study. In regard to pre-processing methods of BOLD signal, we only registered the BOLD images by non-linear transformation. We would improve pre-processing methods to reduce the distorted regions and guarantee the BOLD signal reliability by the 3D reversed phase encoding method in our further study.

## Conclusion

In conclusion, this study applied rs-fMRI and 1H-MRS to detect functional and metabolites changes in the hippocampus of SAE rats. Briefly, we conclude from the present study that alterations in ALFF values and the mI/Cr and Glx/Cr ratios in the hippocampus are negatively related to cognitive function in SAE rats. Furthermore, microglial activation and accompanied inflammation were closely related to brain function during early SAE. Further research will help to improve the understanding of earlier identification of cognitive deficits in SAE.

## Data availability statement

The raw data supporting the conclusions of this article will be made available by the authors, without undue reservation.

## Ethics statement

The animal study was reviewed and approved by the Institutional Animal Care and Use Committee of Nanjing Medical University (IACUC: 2103051).

## Author contributions

HJL, HB, and HSL designed the research. HJL, HSL, YX, and CZ performed the research. HJL, YS, YC, SS, and XX collected and analyzed the data. YS and HB provided the administrative support. HJL, QL, and HB drafted the manuscript. All authors edited and approved the manuscript.

## Footnotes

1
https://www.fil.ion.ucl.ac.uk/spm/

2
http://rfmri.org/dpabi

3
https://resources.mestrelab.com
